# From the Editor's desk

**DOI:** 10.1155/S1463924679000523

**Published:** 1979


rational argument would appear to point away from centralisation; just as sure as the automation of clothes washing first led to big central laundries with large capacity machines, and then to their partial demise following the evolution of the highly efficient home washing machine and community launderette.

*F.L. Mitchell*

### From the Editor's desk

This issue marks a significant point in the Journal of Automatic Chemistry's life, the final issue of the first fornative year, but Volume 1 will be completed with the October issue. Whilst there has been a healthy supply of papers being submitted for consideration in the Journal there has not been a flood of letters from the readership. Those letters we have received express the value of the new journal and they encourage us to continue along the lines established throughout the first year. In general the breakdown of readership is approximately divided equally between those with clinical and those with industrial interests. My own colleagues with an industrial background find considerable use reading all the articles so it is worthwhile not being put off by the title of the paper or by the affiliation of the authors. There is considerable similarity in approach and problem between work within an organisation such as the Clinical Research Centre and work relating to the analysis of tobacco smoke at the Laboratory of the Goverment Chemist. There is a great deal to be gained by communications across disciplines – it is too easy to say that there is far too much to read on one's own subject area. However, there is a common interest in instrumentation and it is vital that the advantages of microprocessors and new technical developments are integrated correctly into our working lives. This I submit, requires a collective objective.

In his editorial commentary Dr. Mitchell discusses the shift from centralised power to local control. This is a very important change, however it does not detract from the value that can be gained by a centralised facility, but it does more easily put the control where it ought to be. Any user of a computer system wants to feel that he is in complete control. How this is achieved is not important but many of the centralised approaches in the past have failed to meet this objective. Successful manufacturers are producing instruments more in line with the market needs. The control of the instrument must be situated with the instrument. However, it is vitally important that such instruments are able to communicate with each other and with a central intelligence. The first attempts by Hewlett Packard to introduce microprocessor technology into their automated gas chromatographs did not achieve a great degree of acceptance, they were unable to talk simply to other computers. The second generation from the same company has not a similar fault, is expandable and represents a significant advance in the first attempt. With such advantages there are really two problems that must be overcome. It is important to standardise interfaces so that it is a relatively simple matter of a connection to other instruments or computers. There is also a need to generate truly portable software packages. Software is a subject which is generally glossed over in the literature but it is a very costly item which must be integrated into the cost benefit equation. Many instrument manufacturers absolve themselves from this discussion by offering a basic computer on the grounds that everybody will be able to 'speak' basic in the near future.

The paper by Professor Bonner Denton published in the third issue of the Journal of Automatic Chemistry attempts to overcome one part of the problem, that of control. The availability of cheap development computers such as the Commodore PET is also seen by some as a way out of the problem. However, in my own experience, whilst the hardware is generally a quantifiable aspect of the problem, the software in terms of specification and production is both more expensive and difficult to obtain in acceptable timescales.

At the 3rd European Congress of Clinical Chemistry at Brighton, (a report of this appears on page 226), I was able to hold an editorial meeting with my clinical corresponding editors who had assembled for the meeting. This was of considerable value and many useful suggestions helpful to the progress of the Journal emerged. One important point was that the meeting complemented the face to face meeting I have had with each member of the editorial team. This will make it much easier in future to correspond between ourselves and will I am sure increase the flow of information. Perhaps the most useful point that emerged is that Dr. Collombel suggested that a glossary of published evaluation reports should be published in the Journal on a continuing basis. A retrospective list of all instrument evaluations currently available will be published and periodically will be updated. In conjunction with the references a short abstract of the evaluation will be provided.

Clinical chemists place considerable value on evaluation reports, and have established a protocol for such evaluations. In industrial applications such reports are not in vogue. They are often confined to intra-organisational reports and not published in the scientific literature. However, with considerable pressure on available resources it is hoped that some standardisation of evaluation report in the industrial market can emerge. Certainly it seems that this is one area where industrial chemists can learn from our clinical colleagues. One useful role of the Journal is to publish evaluation reports and to do this in a quick timescale. It is an important factor in the communication of information from manufacturers to users and vice versa.

*Peter B. Stockwell*

